# Highly Efficient Electrocatalytic Uranium Extraction from Seawater over an Amidoxime‐Functionalized In–N–C Catalyst

**DOI:** 10.1002/advs.202201735

**Published:** 2022-06-17

**Authors:** Xiaolu Liu, Yinghui Xie, Mengjie Hao, Zhongshan Chen, Hui Yang, Geoffrey I. N. Waterhouse, Shengqian Ma, Xiangke Wang

**Affiliations:** ^1^ College of Environmental Science and Engineering North China Electric Power University Beijing 102206 P.R. China; ^2^ MacDiarmid Institute for Advanced Materials and Nanotechnology School of Chemical Sciences The University of Auckland Auckland 1142 New Zealand; ^3^ Department of Chemistry University of North Texas Denton TX 76201 USA

**Keywords:** adsorption–electrocatalysis, indium–nitrogen–carbon, mechanism, seawater, uranium extraction

## Abstract

Seawater contains uranium at a concentration of ≈3.3 ppb, thus representing a rich and sustainable nuclear fuel source. Herein, an adsorption–electrocatalytic platform is developed for uranium extraction from seawater, comprising atomically dispersed indium anchored on hollow nitrogen‐doped carbon capsules functionalized with flexible amidoxime moieties (In–N_*x*_–C–R, where R denotes amidoxime groups). In–N_*x*_–C–R exhibits excellent uranyl capture properties, enabling a uranium removal rate of 6.35 mg g^−1^ in 24 h, representing one of the best uranium extractants reported to date. Importantly, In–N_*x*_–C–R demonstrates exceptional selectivity for uranium extraction relative to vanadium in seawater (8.75 times more selective for the former). X‐ray absorption spectroscopy (XAS) reveals that the amidoxime groups serve as uranyl chelating sites, thus allowing selective adsorption over other ions. XAS and in situ Raman results directly indicate that the absorbed uranyl can be electrocatalytically reduced to an unstable U(V) intermediate, then re‐oxidizes to U(VI) in the form of insoluble Na_2_O(UO_3_·H_2_O)*_x_* for collection, through reversible single electron transfer processes involving InN*_x_* sites. These results provide detailed mechanistic understanding of the uranium extraction process at a molecular level. This work provides a roadmap for the adsorption–electrocatalytic extraction of uranium from seawater, adding to the growing suite of technologies for harvesting valuable metals from the earth's oceans.

## Graphical abstract

Porous indium−nitrogen−carbon capsules functionalized with flexible amidoxime groups enable highly efficient uranium extraction from seawater. The flexible amidoxime groups allow selective uranyl adsorption, while the InN*_x_* single atom sites provide a reversible single electron transfer platform for uranyl conversion to a harvestable Na_2_O(UO_3_·H_2_O)*_x_* precipitate via a U(V) intermediate.

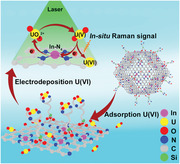

## 1 Introduction

Humans rely heavily on fossils fuels for energy and transportation. This dependence is not sustainable and is the main cause of global warming, motivating a switch to renewable and clean energy sources. The Paris Agreement aims to lower net carbon dioxide emissions to preindustrial levels over the next 30 years.^[^ ^1^ ^]^ Nuclear power has been long been pursued as an alternative to polluting fossil fuel energy.^[^ ^2^ ^]^ This technology utilizes enriched uranium as a fuel source in nuclear fission reactors. However, conservative estimates suggest uranium reserves on land to be around 7.6 million metric tons, sufficient to feed existing nuclear power plants for less than 100 years. This represents a serious bottleneck to the utilization of nuclear power as part of a zero‐carbon energy infrastructure. However, ≈4.5 billion metric tons of uranium is present in the earth's oceans, representing a rich uranium resource that would enable sustainable nuclear energy generation for thousands of years if it could be efficiently harvested.^[^ ^3^ ^]^ Thus, the discovery of technologies that allow efficient and selective uranium capture from seawater is of great practical significance for the nuclear energy sector.

From a practical viewpoint, selective capture of uranium from seawater is highly challenging because of the ultralow uranium concentration in seawater (≈3.3 ppb), interference from coexisting ions, and the complicated biological environment that can passivate the sorbents and extraction devices.^[^ ^3^ ^]^ To address these issues, extraction technologies with high selectivity, fast removal kinetics, and a large capacity towards for uranium removal are highly sought after. Direct adsorption technologies have been widely pursued for uranium extraction, using adsorbents such as porous carbons,^[^ ^4^ ^]^ metal–organic frameworks,^[^ ^5^ ^]^ covalent organic frameworks,^[^ ^6^ ^]^ biomaterials,^[^ ^7^ ^]^ and porous polymers.^[^ ^8^ ^]^ These adsorbents typically contain functional groups with a high affinity for uranyl ions. However, this traditional adsorption approach has serious limitations, since uranyl binding blocks chelating sites and thus limits the uranium uptake/removal capacity, whilst introducing further technical challenges around uranium recovery and adsorbent recycling. A further drawback is the rapid adsorption capacity losses due to passivation or corrosion by marine bacteria and algae in seawater.

Electrochemical methods are emerging as an attractive alternative to simple sorption technologies for selective uranium extraction from seawater and other aqueous systems. Electrochemical methods offer the advantages of accelerated migration rates of uranyl ions, excellent resistance to marine microbial corrosion, large extraction capacities, cost effectiveness and energy efficiency.^[^ ^8^, ^9^ ^]^ Recently we reported an iron–nitrogen–carbon (Fe–N_*x*_–C)‐based adsorption–electrocatalysis strategy for efficient uranium extraction from seawater.^[^ ^10^ ^]^ A Fe–N_*x*_–C–R electrocatalyst functionalized with amidoxime groups (R) enabled the selective capture of uranyl from spiked seawater and natural seawater samples, culminating in the formation of easily collected yellow Na_2_O(UO_3_·H_2_O)*_x_* precipitates via reversible electron transfer processes (U^6+^ ↔ U^5+^) involving the N‐doped carbon‐supported FeN_4_ single atom sites. Whilst this approach was highly novel, the uranium extraction capacity of the Fe–N_*x*_–C–R adsorbent–electrocatalyst was a modest ≈1.2 mg g^−1^ d^−1^ in natural seawater, motivating further research aimed at increasing the uranium uptake capacity. Meanwhile, better understanding of the catalytic mechanism for uranium extraction would allow optimization of electrocatalyst design.

Inspired by our previous work,^[^ ^10^ ^]^ we present herein a novel functionalized indium–nitrogen–carbon catalyst (In–N*_x_*–C–R), comprising atomically dispersed indium sites dispersed over hollow N‐doped porous carbon capsules (In–N*_x_*–C) postsynthetically functionalized with flexible surface amidoxime groups (R). The flexible amidoxime groups enabled selective uranyl adsorption. Subsequently, the InN*_x_* sites enabled rapid electrocatalytic conversion of the adsorbed U(VI) ions to solid Na_2_O(UO_3_·H_2_O)*_x_* through a reversible U^6+^ ↔ U^5+^ redox pathway, thereby delivering a remarkably high uranium extraction capacity of 6.35 mg g^−1^ d^−1^ from natural seawater, surpassing most state‐of‐the‐art adsorbents. Moreover, In−N*_x_*−C−R showed outstanding selectivity for uranium relative to vanadium at high ionic strengths (8.75 times more selective towards uranium), thus overcoming one of the key obstacles in selective uranium extraction from seawater. Furthermore, using an array of complementary experimental techniques, the mechanistic steps underpinning uranyl conversion to Na_2_O(UO_3_·H_2_O)*_x_* over In−N*_x_*−C−R were elucidated. X‐ray absorption spectroscopy (XAS) together with in situ Raman spectroscopy confirmed the formation of the U(V) intermediate during the electrochemical redox processes, allowing the electrochemical uranium extraction mechanism on the In−N*_x_*−C−R catalyst surface to be fully understood at a molecular level. Results lay a blueprint for the future design of large‐scale adsorption−electrocatalytic systems for uranium extraction from seawater.

## 2 Results and Discussion

### 2.1 Synthesis and Characterization of Functionalized Indium−Nitrogen−Carbon Adsorption−Electrocatalyst

**Figure 1:**
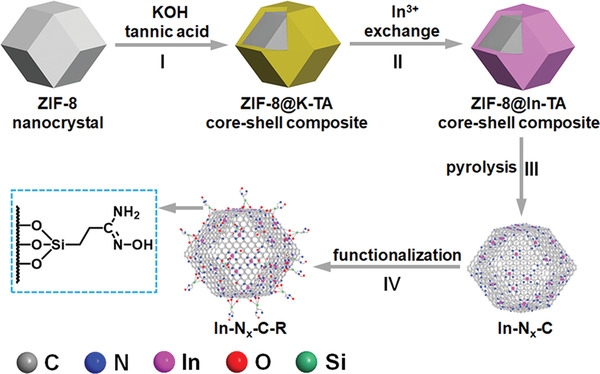
Schematic illustration of the synthesis of In−N*_x_*−C, and its further functionalization with flexible amidoxime groups to produce In−N*_x_*−C−R. Hydrogen atoms have been omitted for clarity.

**Figure 2:**
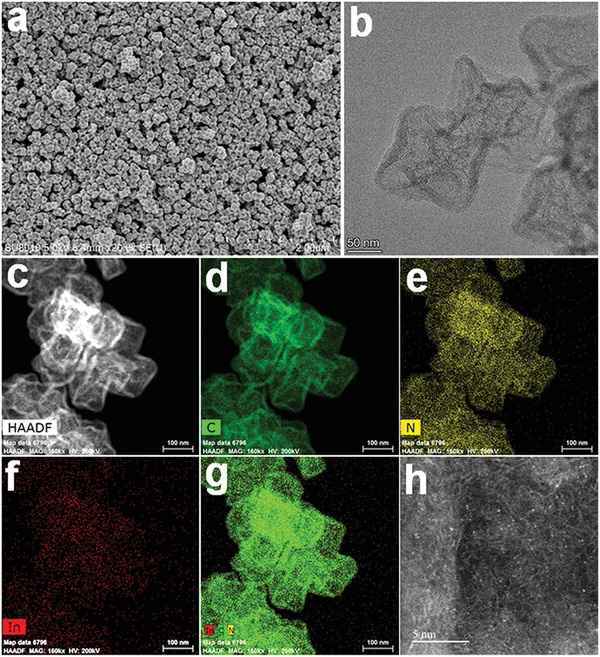
a,b) SEM and TEM images of In−N*_x_*−C. c−g) HAADF‐STEM image and corresponding EDS maps reveal a homogeneous distribution of C (green), N (yellow), and In (red) over the carbon capsules. h) Aberration‐corrected HAADF‐STEM image of In−N*_x_*−C, showing the atomically dispersed indium. The scale bars represent a) 2 µm, b) 50 nm, c−g) 100 nm, and h) 5 nm.

The stepwise synthesis of the functionalized indium−nitrogen−carbon catalyst (In−N*_x_*−C−R, where R represents the flexible amidoxime groups) is shown in **Figure** **1**. Key steps include i) uniform coating of ZIF‐8 crystals with a potassium−tannic acid (K−TA) polymer to create a ZIF‐8@K−TA core−shell composite (step I).^[^ ^11^ ^]^ ii) replacement of the potassium cations in the K−TA shell with In(III) cations to obtain ZIF‐8@In−TA (step II). ZIF‐8@In−TA contained 1.45 wt% indium by inductively coupled plasma optical emission spectrometry (ICP−OES, Table S2, Supporting Information) analysis. Powder X‐ray diffraction (PXRD), scanning electron microscopy (SEM), transmission electron microscopy (TEM), high‐angle annular dark‐field scanning transmission electron microscopy (HAADF‐STEM), and corresponding energy‐dispersive X‐ray spectroscopy (EDS) mapping images showed the initial crystallinity and the dodecahedral morphology of the ZIF‐8 core were retained after metal−tannic acid shell coating step (Figures S1, S4−S7, Supporting Information). The shell thickness was around 12 nm (Figure S7a,b, Supporting Information). (iii) Pyrolysis of ZIF‐8@In−TA under an argon atmosphere yielded atomically dispersed InN*_x_* immobilized on porous N‐doped carbon capsules (In−N*_x_*−C, step III). SEM and TEM images clearly showed the hollow polyhedral morphology of In−N*_x_*−C (**Figure** **2**a,b). No large indium‐containing particles or aggregates were observed, suggesting that indium atoms were independently anchored on the hollow capsules. Low magnification HAADF‐STEM and corresponding EDS mapping images revealed uniform distributions of In and N over the hollow carbon architecture (Figure 2c–g). PXRD showed characteristic peaks at ≈21.9° and ≈43.6°, which could readily be ascribed to the (002) and (100) reflections of graphitic carbon domains (Figure S2, Supporting Information). No metallic indium or oxide crystalline peaks were observed, consistent with the electron microscopy results. Spherical‐aberration‐corrected HAADF‐STEM imaging enabled detailed structural characterization of In−N*_x_*−C. The bright spots in Figure 2h showed that indium was atomically over the hollow N‐doped carbon capsules.

**Figure 3:**
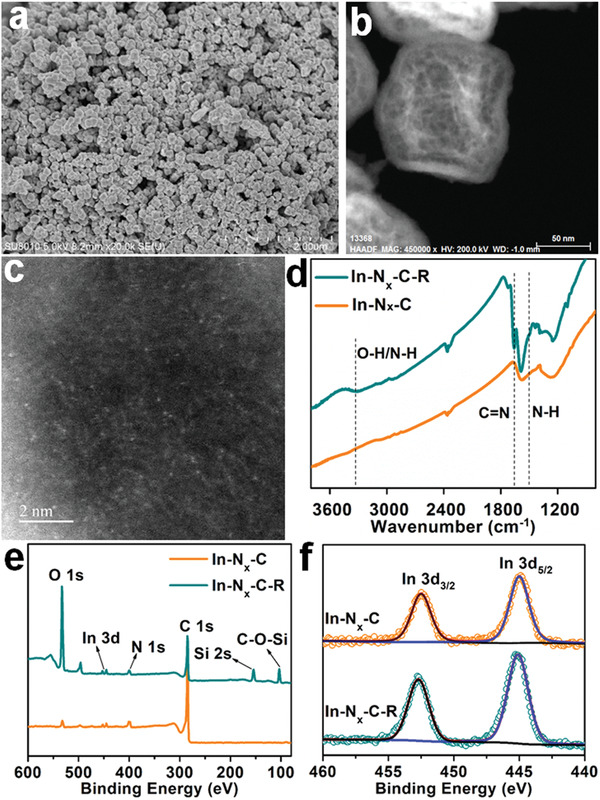
a,b) SEM and HAADF‐STEM images of In−N*_x_*−C−R. c) Aberration‐corrected HAADF‐STEM image of In−N*_x_*−C−R, showing the atomically dispersed indium. The scale bars represent a) 2 µm, b) 50 nm, and c) 2 nm. d) FT‐IR spectra for In−N*_x_*−C and In−N*_x_*−C−R. e) XPS spectra of In−N*_x_*−C and In−N*_x_*−C−R. f) In 3d XPS spectra of In−N*_x_*−C and In−N*_x_*−C−R.

Amidoxime functional groups demonstrate a strong binding affinity for uranyl through cooperative interactions, thus leading to their utilization in adsorbents for selective uranium extraction from seawater.^[^ ^12^ ^]^ Accordingly, the final step in the synthesis of In−N*_x_*−C−R was the introduction of amidoxime functional groups on the surface of the In−N*_x_*−C capsules (step IV, Figure 1). To achieve this, In−N*_x_*−C was treated in a nitric acid/sulfuric acid mixture to introduce surface −COOH/−OH groups,^[^ ^13^ ^]^ then refluxed with (2‐cyanoethyl)triethoxysilane to introduce cyano moieties^[^ ^14^ ^]^ (Figure S8, Supporting Information). Cyano groups were then transformed into amidoxime groups through a hydrolysis reaction.^[^ ^6^, ^8^ ^]^ Thus, a final treatment with hydroxylammonium chloride in ethanol produced In−N*_x_*−C−R, with flexible amidoxime groups (R) on the surface of the hollow indium−nitrogen−carbon capsules (Figure 1, step IV).^[^ ^6^, ^8^ ^]^ PXRD, SEM and HAADF‐STEM images demonstrated that In−N*_x_*−C−R retained the structure and hollow morphology of In−N*_x_*−C (**Figure** **3**a,b and Figure S3, Supporting Information). Spherical‐aberration‐corrected HAADF‐STEM imaging confirmed that the atomically dispersed indium sites were still firmly anchored on the hollow N‐doped carbon capsules after amidoxime introduction (Figure 3c). Fourier transform infrared spectroscopy (FT‐IR) of In−N*_x_*−C−R showed new peaks at ≈3100−3300, 1635, and 1572 cm^−1^ corresponding to −OH, C═N, and N−H vibrations of amidoxime groups, respectively (Figure 3d).^[^ ^9^, ^15^ ^]^ The elemental composition of In−N*_x_*−C−R was explored by X‐ray photoelectron spectroscopy (XPS) spectroscopy. The XPS spectra of In−N*_x_*−C−R showed the presence of C, N, Si, O, and In (Figure 3e). The observation of the Si 2p signal around 102.8 eV, due to C−O−Si, confirmed the successful R group functionalization. The In 3d_5/2_ and 3d_3/2_ binding energies of 445.1 eV and 452.7 eV, respectively, (Figure 3f), were situated between values for indium metal and In_2_O_3_, suggesting a cationic In*^*δ*^* ^+^ (0 < *δ* < 3) species in In−N*_x_*−C and In−N*_x_*−C−R (most likely In^3+^ coordinated by N).^[^ ^16^ ^]^ The C 1s spectrum of In−N*_x_*−C−R was similar to that of In−N*_x_*−C, with the exception being the appearance of an enhanced signal at 287.9 eV due to the oxidized carbon species (C = O bonds) (Figures S9 and S11, Supporting Information). Four types of N environments were found in the In−N*_x_*−C and In−N*_x_*−C−R samples, including pyridinic−N, pyrrolic−N, graphitic−N, and N−O*_x_* moieties (Figures S10 and S12, Supporting Information). Pyridinic and pyrrolic nitrogen atoms in the carbon shells acted to anchor the indium cations via coordination bonds.^[^ ^17^ ^]^

**Figure 4:**
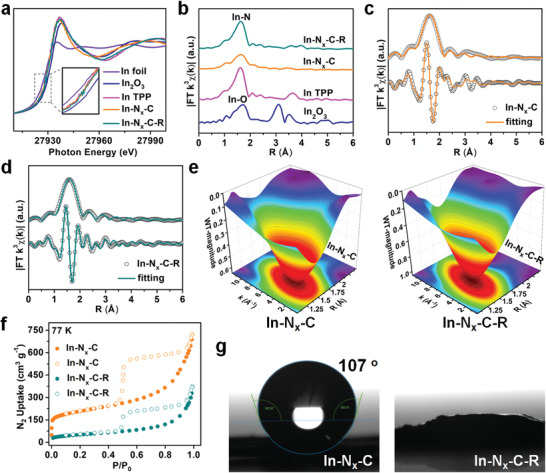
a) In K‐edge XANES spectra. b) FT k^3^‐weighted *χ*(k) function of EXAFS spectra. c) EXAFS fitting curve for In−N*_x_*−C. d) EXAFS fitting curve for In−N*_x_*−C−R. e) WT contour plots for In−N*_x_*−C and In−N*_x_*−C−R. f) N_2_ sorption isotherms for In−N*_x_*−C and In−N*_x_*−C−R. g) Contact angles for water droplets on pressed pellets of In−N*_x_*−C and In−N*_x_*−C−R.

To further investigate the local coordination of indium cations in the materials, X‐ray absorption structure (XAS) analyses were performed. In K‐edge X‐ray absorption near edge structure (XANES) spectra showed the pre‐edges of In−N*_x_*−C and In−N*_x_*−C−R were close to data for indium(III) tetraphenyl porphyrin (In TPP), indicating that the isolated indium atoms likely possessed a square−planar InN_4_ configuration (**Figure** **4**a). The absorption edges for In−N*_x_*−C and In−N*_x_*−C−R were located between data for In foil and In_2_O_3_ (and closer to the latter) suggesting the In*^*δ*^* ^+^ (0 < *δ* < 3). Nitrogen is less electronegative than oxygen, thus the edge position for In^3+^ in the samples was not expected to match the In^3+^ reference sample (In_2_O_3_) exactly. The Fourier transformed extended X‐ray absorption fine structure (FT‐EXAFS) spectra of In−N*_x_*−C and In−N*_x_*−C−R exhibited prominent peaks at around 1.63 Å, showing oscillations similar to those of In TPP, further evidence for the presence of atomically dispersed porphyrin‐like InN_4_ sites (Figure 4b). The excellent fitting results confirmed a InN_4_ coordination (Figure 4c,d, Table S1, Supporting Information). In addition, we carried out the EXAFS wavelet transform (WT) analyses to gain in‐depth information about the indium species in the materials. Figure 4e and Figure S13 (Supporting Information) show the WT contour plots of In−N*_x_*−C, In−N*_x_*−C−R, In foil, In_2_O_3_, and In TPP. As expected, In−N*_x_*−C, and In−N*_x_*−C−R exhibited only one intensity maximum around 4.0 Å^−1^ in k space (≈1.63 in R space) associated with In−N contributions (the same as In TPP in Figure S13, Supporting Information), suggesting that the indium atoms existed as mononuclear centers rather than clusters or nanoparticles (Figure 4e). For comparison, In foil and In_2_O_3_ showed intensity maxima at ≈4.51 Å^−1^ (≈2.89 in R space) and ≈4.55 Å^−1^ (≈1.61 in R space) in k space, respectively, corresponding to In−In bonds and In−O bonds, respectively (Figure S13, Supporting Information). These results further confirmed that the InN_4_ sites in In−N*_x_*−C were unaffected by functionalization with flexible amidoxime groups in the creation of In−N*_x_*−C−R. The simultaneous existence of well‐dispersed InN_4_ catalytic active sites and flexible amidoxime uranyl binding groups was expected to impart In−N*_x_*−C−R with excellent properties for the adsorption−electrocatalytic extraction of uranium from seawater.

### 2.2 Physiochemical Characterization

In−N*_x_*−C and In−N*_x_*−C−R contained 0.48 and 0.47 wt% of indium, respectively, as determined by inductively coupled plasma mass spectroscopy (ICP‐MS) (Table S2, Supporting Information). Elemental analyses established the nitrogen contents in the samples to be 6.77 and 7.75 wt%, respectively (Table S2, Supporting Information). The Raman spectra for In−N*_x_*−C and In−N*_x_*−C−R exhibited characteristic D‐band (1350 cm^−1^) and G‐band (1590 cm^−1^) signals due to disordered (sp^3^) and graphitic (sp^2^) carbon, respectively (Figure S14, Supporting Information). Nitrogen adsorption−desorption isotherms were carried out to probe the accessible porosity of these materials at 77 K. The adsorption isotherms showed rapid N_2_ uptake at low partial pressures (*P*/*P*_0_< 0.1) and increased staged uptake at pressures between 0.1 < *P*/*P*_0_ < 0.95, suggesting that both micropores and mesopores were present in the In−N*_x_*−C and In−N*_x_*−C−R samples (Figure 4f, Figure S15, Supporting Information). Large adsorption−desorption hysteresis loops were observed at partial pressures (*P*/*P*_0_) between 0.5 and 0.95, indicating the existence of mesopores in both catalysts. The calculated Brunauer−Emmett−Teller (BET) surface area of In−N*_x_*−C−R was ≈183 m^2^ g^−1^, much lower than that of In−N*_x_*−C (701 m^2^ g^−1^). Results suggest that the functional amidoxime groups in In−N*_x_*−C−R occupied some of the pore space in the N‐doped carbon framework. Water contact angle experiments were further conducted to probe the surface wettability of In−N*_x_*−C and In−N*_x_*−C−R. A water contact angle of 107° was measured for In−N*_x_*−C indicating considerable hydrophobicity, whereas the water droplet was rapidly absorbed by In−N*_x_*−C−R (Figure 4g). These results demonstrated that the flexible amidoxime groups significantly improved the hydrophilicity of the hollow N‐doped carbon capsules. The electrical resistance of In−N*_x_*−C−R was measured to be slightly higher than that of In−N*_x_*−C, indicating that excellent conductivity was retained after functionalization with amidoxime groups (Figure S16, Supporting Information). In view of the above structural and physical attributes, In−N*_x_*−C−R offered many desirable attributes as an adsorption−electrocatalyst for uranium extraction from seawater.

### 2.3 Uranium Extraction Studies

**Figure 5:**
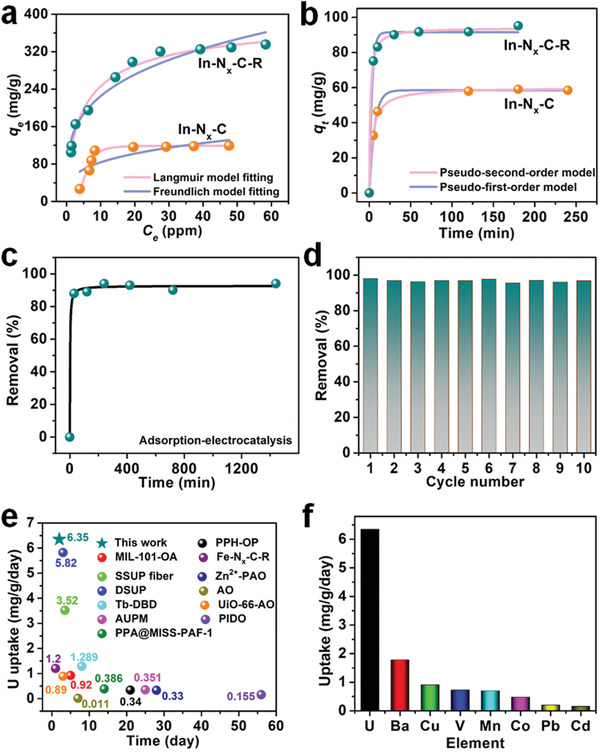
a) Uranium adsorption isotherms for In−N*_x_*−C and In−N*_x_*−C−R. b) Uranium adsorption kinetics of In−N*_x_*−C and In−N*_x_*−C−R at an initial uranium concentration of 10 ppm (the pH of the uranium‐spiked seawater solutions were adjusted to ≈8 using Na_2_CO_3_). c) Uranium extraction from uranium‐spiked seawater with an initial uranium concentration of 10 ppm, using In–N*_x_*–C–R as an adsorbent–electrocatalyst (the pH of the uranium‐spiked seawater solutions were adjusted to ≈8 using Na_2_CO_3_). d) Recyclability of In−N*_x_*−C−R for uranium extraction from uranium‐spiked seawater. e) Comparison of uranium extraction uptake performance of In−N*_x_*−C−R and other reported materials in natural seawater. f) Specificity of In−N*_x_*−C−R for uranium extraction from natural seawater.

We first tested the adsorption performance of In−N*_x_*−C and In−N*_x_*−C−R in uranium‐spiked seawater solutions. The uptake capacity, kinetic data, and fitting results are summarized in Tables S3 and S4 (Supporting Information). In−N*_x_*−C−R showed a very high adsorption capacity of 335.4 mg g^−1^ (in the uranium concentration range of 0−100 ppm) at a sorbent‐to‐solution ratio of 0.1 g L^−1^ (**Figure** **5**a). Moreover, In−N*_x_*−C−R also possessed extremely rapid kinetics, achieving a removal percentage of 90% in only 30 min (Figure 5b). In comparison, In−N*_x_*−C showed a much lower uranium uptake capacity and lower adsorption efficiency under similar conditions. The flexible amidoxime groups of In−N*_x_*−C−R clearly enhanced uranium uptake. Based on the excellent adsorption properties and conductivity of In−N*_x_*−C−R, we next attempted electrocatalytic uranium extraction tests from spiked seawater and natural seawater. The experimental tests were performed using a square wave conversion method (employing alternating voltages between −5 V and 0 V).^[^ ^9^, ^10^ ^]^ In−N*_x_*−C−R was loaded onto carbon felt at a loading of 5 mg cm^−3^ to form the working electrode. At this loading, ≈94% (≈200 mg g^−1^) of the uranium in a ≈10 ppm spiked seawater solution was removed in 240 min (Figure 5c). In comparison, this adsorption−electrocatalytic process delivered a uranium uptake capacity ≈2.1 times higher than simple physicochemical adsorption methods. An extraction rate of >94% was maintained over ten extraction cycles, confirming the durability of In−N*_x_*−C−R as a promising adsorption−electrocatalyst for practical uranium extraction (Figure 5d). Next, we evaluated the uranium extraction performance of In−N*_x_*−C−R in natural seawater. As expected, In−N*_x_*−C−R delivered an impressive uranium extraction capacity of 12.7 mg g^−1^ in 2 d (6.35 mg g^−1^ d^−1^), comparable to the best adsorbents reported so far uranium removal from natural seawater (as seen in Figure 5e, Table S5, Supporting Information).^[^ ^7^, ^8^, ^10^, ^18^ ^]^ In−N*_x_*−C−R thus possesses many benefits for real world applications. It is well known that vanadium (as the vanadyl ion) is the main cationic competitor in uranium extraction from seawater when using amidoxime‐based groups for uranyl binding. We thus studied the selectivity of In−N*_x_*−C−R towards uranium over other metal ions that exist in seawater. As shown in Figure 5f, the adsorption capacity to the uranium of In−N*_x_*−C−R was 8.75 times higher than that of vanadium after adsorption−electrocatalytic processing, suggesting good potential applicability for large−scale seawater uranium extraction. We estimated the cost of synthesizing In−N*_x_*−C−R to be ≈$41 USD g^−1^, suggesting the economic feasibility of the catalyst. Moreover, the cost for uranium extraction (to produce 1 kg of Na_2_O(UO_3_·H_2_O)*_x_*) using In−N*_x_*−C−R was estimated to be ≈$806 USD.

### 2.4 Adsorption−Electrocatalytic Uranium Extraction Mechanism Studies

**Figure 6:**
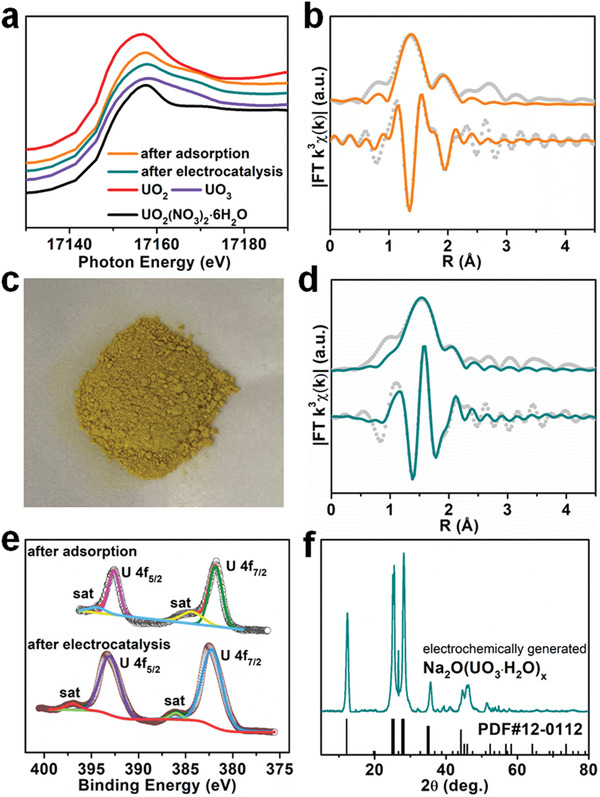
a) A comparison of U L_III_‐edge XANES spectra for In−N*_x_*−C−R after uranium adsorption and electrocatalysis, along with data for various uranium standards. b) EXAFS fitting curve for In−N*_x_*−C−R after adsorption of uranium. c) A photograph of the electrochemically generated product Na_2_O(UO_3_·H_2_O)*_x_*. d) EXAFS fitting curve for In−N*_x_*−C−R after electrocatalysis. e) A comparison of U 4f XPS spectra for In−N*_x_*−C−R after uranium adsorption and electrocatalysis. f) PXRD pattern of the electrochemically generated Na_2_O(UO_3_·H_2_O)*_x_*. The reference data for UO_2_, UO_3_, and UO_2_(NO_3_)_2_·6H_2_O in (a) were taken from our previous work.^[^ ^10^ ^]^

Encouraged by the aforementioned results, we next conducted detailed studies to explore the adsorption−electrocatalysis mechanism used by In−N*_x_*−C−R for uranium extraction. A wide range of complementary techniques, including XAS, XPS, electron microscopy, and in situ Raman spectroscopy were employed for this purpose. U L_III_‐edge XANES spectra revealed that after uranyl adsorption, a spectrum similar to that of the uranyl nitrate hexahydrate standard was obtained, consistent with the presence of surface adsorbed U(VI) (**Figure** **6**a). FT‐EXAFS spectra and their fitting results further revealed the uranium coordination environment was consistent with uranyl−amidoxime *η^2^* binding (Figure 6b, Table S6, Supporting Information).^[^ ^12a^ ^]^

To obtain enough electrocatalytically deposited solid product for further characterization studies, adsorption−electrocatalysis experiments were conducted in a ≈1000 ppm uranium‐spiked seawater solution. The experiments yielded a yellow solid (Figure 6c). U L_III_‐edge XANES spectrum showed the yellow product to contain U(VI) (Figure 6a). The FT‐EXAFS spectrum showed a peak at ≈1.52 Å, which could readily be assigned to a U—O scattering path (Figure 6d). The FT‐EXAFS data was well fitted by a uranium center being coordinated by two axial oxygen atoms and four square planar oxygen atoms with U—O bond lengths of ≈1.8 Å and ≈2.2 Å, respectively (Figure 6d, Table S7, Supporting Information), suggesting the presence of a UO_3_ subunit in the structure. The U 4f XPS spectrum confirmed the presence of U(VI) after both uranyl adsorption and electrocatalysis tests (Figure 6e). No U(IV) species was detected. PXRD identified the yellow product as Na_2_O(UO_3_·H_2_O)*_x_*, consistent with our previous work using a Fe−N*_x_*−C−R adsorbent−electrocatalyst (Figure 6f).^[^ ^10^ ^]^ HAADF‐STEM and EDS element mapping images revealed uniform distribution of C, N, In, Si, O, Na, and U elements in the In−N*_x_*−C−R capsules after adsorption−electrocatalysis (Figure S17, Supporting Information). The hollow morphology was retained indicating good catalyst robustness. TEM and HRTEM images showed the generation of yellow solid particles attached to the hollow capsules with an interplanar spacing of ≈2.8 Å (Figure S18, Supporting Information), corresponding to the (002) reflections of Na_2_O(UO_3_·H_2_O)*_x_*. Control experiments were further carried out with deionized water and sodium chloride solution, respectively. No solid product was produced in the absence of sodium ions, whilst the yellow Na_2_O(UO_3_·H_2_O)*_x_* precipitate was generated in the sodium chloride solution (Figures S19 and S20, Supporting Information). FT‐IR spectroscopy further revealed the chemical structure of Fe−N*_x_*−C−R was retained after cycling tests (Figure S21, Supporting Information).

**Figure 7:**
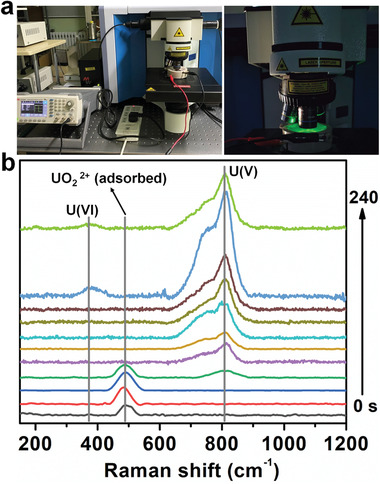
a) Photographs showing the in situ electrochemical−Raman microscope system. b) In situ Raman spectra collected from the In−N*_x_*−C−R/carbon felt working electrode in uranium‐spiked seawater during the adsorption−electrocatalysis process.

To further investigate the formation of the Na_2_O(UO_3_·H_2_O)*_x_* precipitate, in situ Raman spectra were collected from In−N*_x_*−C−R in uranium‐spiked seawater during the adsorption−electrocatalysis process (**Figure** **7**a,b and Figure S22, Supporting Information). Before the electrocatalysis, a Raman signal due to adsorbed uranyl was observed at 489 cm^−1^, suggesting the flexible amidoxime functional groups had bound some U(VI) from the spiked−seawater solution.^[^ ^9a^ ^]^ During the square wave potential cycling, a U(V) signal at 810 cm^−1^ appeared, indicating that adsorbed uranyl were reduced to U(V) intermediate.^[^ ^19^ ^]^ Subsequently, the signal intensity of adsorbed uranyl decreased and then disappeared completely, indicating the complete transformation of adsorbed uranyl to U(V) intermediate. No U(IV) or U(VI) signals were observed with time. A new peak appeared at 374 cm^−1^ appeared in the spectra at ≈240 s. We speculated this feature arises from the oxidation of unstable U(V) to U(VI) in the presence of sodium ions, forming the Na_2_O(UO_3_·H_2_O)*_x_* precipitate.

The results here demonstrate that hollow functionalized indium−nitrogen−carbon capsules (In−N*_x_*−C−R) efficiently adsorb uranyl, which are then electrocatalytically reduced to a U(V) intermediate. During square wave potential cycling, re‐oxidation of the U(V) to U(VI) yields a solid precipitate (i.e., Na_2_O(UO_3_·H_2_O)*_x_*) via a reversible electron transfer process. The outstanding uranium extraction properties of In−N*_x_*−C−R can be attributed to: i) the conductive hollow capsule structure which allows the efficient mass and electron transfer processes; ii) the flexible amidoxime functional groups which provide a high affinity and selectivity for uranyl relative to other competing ions; iii) the abundance of well dispersed InN*_x_* sites for electron transfer and electrocatalytic redox reactions; iv) the precipitation of collectible Na_2_O(UO_3_·H_2_O)*_x_* in the presence of sodium ions.

## 3 Conclusion

In summary, flexible amidoxime group‐functionalized indium−nitrogen−carbon capsules were developed as a highly efficient adsorption−electrocatalyst system for uranium extraction from seawater. The extraction capacity of In−N*_x_*−C−R is as high as 6.35 mg g^−1^ d^−1^ in natural seawater. In situ Raman and XAS measurements allowed the extraction mechanism to be fully understood at the molecular level, involving the transformation of aqueous uranyl to Na_2_O(UO_3_·H_2_O)*_x_* precipitates in an adsorption−electrocatalysis process with a U(V) intermediate. Our work reveals that adsorption−electrocatalyst systems have many advantages over direct adsorbents for uranium extraction from seawater, such as higher uranium uptake, faster kinetics and facile product recovery. Results lay a firm foundation towards practical systems for sustainable uranium extraction from seawater using renewably generated electricity.

## Conflict of Interest

The authors declare no conflict of interest.

## Supporting information

Supporting information

## Data Availability

The data that support the findings of this study are available from the corresponding author upon reasonable request
