# Supplementary material for: Highly Efficient Electrocatalytic Uranium Extraction from Seawater over an Amidoxime‐Functionalized In–N–C Catalyst
Source: Adv Sci (Weinh). 2022 Jun 17;9(23):2201735. doi: 10.1002/advs.202201735 (PMC9376814; doi:10.1002/advs.202201735)
Supplement: Supplementary file 1 — Supporting information [file ADVS-9-2201735-s001.pdf]

Supporting Information

Highly Efficient Electrocatalytic Uranium Extraction from Seawater over an Amidoxime−Functionalized In−N−C Catalyst

Xiaolu Liu, Yinghui Xie, Mengjie Hao, Zhongshan Chen, Hui Yang, Geoffrey I. N. Waterhouse, Shengqian Ma,* and Xiangke Wang**

Chemicals and instrumentation

All chemicals were sourced from commercial suppliers and used without further purification. The seawater was collected in Maoming, Guangdong Province, China. Powder X−ray diffraction (PXRD) patterns were collected on a Rigaku SmartLab SE X−ray diffractometer equipped with a Cu Kα source. BET surface areas were determined from N_2 adsorption/desorption isotherms collected at 77 K using a Micromeritics TriStar II. Scanning electron microscopy (SEM) images were recorded on a Hitachi SU 8010 Scanning Electron Microscope. Transmission electron microscopy (TEM) images, high−resolution TEM (HRTEM) images, high−angle annular dark−field scanning transmission electron microscopy (HAADF−STEM) images, energy dispersive X−ray spectroscopy (EDS) elements maps, and spherical aberration corrected HAADF−STEM images were recorded on JEOL JEM−2100F or JEM−ARM200F transmission electron microscopes operating at an accelerating voltage of 200 kV. Inductively coupled plasma mass spectrometry (ICP−MS) analyses were performed on Agilent 7800 spectrometer and Perkin Elmer (Optima 5300DV) systems. Inductively coupled plasma optical emission spectrometry (ICP−OES) analyses were performed on a Shimadzu ICPE−9800 system. High−resolution Raman and *in situ* Raman spectra were obtained from powder samples or Fe-N-C-R electrodes on Jobin Yvon HR−800 Raman spectrometer equipped with a cobalt samba single−mode 514 nm diode laser. X−ray photoelectron spectroscopy (XPS) analyses were performed using a Thermo Scientific ESCALAB 250Xi spectrometer, equipped with a monochromatic Al K_α X−ray source. Fourier transform infrared spectra (FT−IR) were recorded on a SHIMADZU IRTracer−100. Cyclic voltammograms (CV) were recorded on a Metrohm Autolab electro−workstation. Electrochemical impedance spectroscopy (EIS) tests were conducted on a CHI 760 electrochemical workstation. Electrochemical uranium adsorption experiments were performed using an UNI−T UTG1005A Function/Arbitrary Waveform Generator. U L−edge X−ray absorption spectroscopy (XAS) data were collected in transmission mode at the Shanghai Synchrotron Radiation Facility (14 W station, SSRF). In K−edge XAS data were collected in transmission mode at SPring−8 (Japan).

Experimental procedures

Synthesis of ZIF−8 nanocrystals

ZIF−8 nanocrystals were synthesized using a reported procedure with a slight modification.[1] In a typical synthesis, 4.1 g of 2−methylimidazole was dissolved in 60 mL of methanol (MeOH) to form a clear solution. 1.68 g of $Zn(NO_3)_2·6H_2O$ was then added into the 2-methylimidazole solution followed by vigorous stirring for 1 h. The mixture was then incubated at room temperature without stirring. After 24 h, the product was isolated as a white powder by centrifugation and washed several times with MeOH, and finally dried overnight under vacuum.

Synthesis of ZIF−8@K−TA

In a typical synthesis, 300 mg of ZIF−8 nanocrystals were dispersed in deionized water by sonication for 10 min. Next, a tannic acid (24 mM, 10 mL) solution of pH=8 (adjusted by adding aqueous 6 M KOH solution) was added to the ZIF−8 dispersion under constant stirring. After stirring for 5 min, the solid product was collected by centrifugation, washed several times with deionized water and MeOH, yielding ZIF−8@K−TA.

Synthesis of ZIF−8@In−TA

50 mg of indium nitrate hexahydrate ($In(NO_3)_2·6H_2O$) was dissolved in 150 mL of MeOH under stirring for 10 min. Subsequently, the ZIF−8@K−TA was added to the indium solution, and the resulting dispersion stirred for 3 h at room temperature. The solid was collected by centrifugation and washed several times with MeOH. Finally, the product was dried in an oven at 40 ^oC under vacuum to yield ZIF−8@In−TA.

Synthesis of In−N_x−C

ZIF−8@In−TA was placed in a tube furnace and heated to 900 °C at a heating rate of 3 °C/min under an Ar atmosphere. After annealing for 3 h at 900 °C, the product was cooled to room temperature under Ar to give In−N_x−C.

Synthesis of In−N_x−C−R

In−N_x−C (200 mg) was kept in a nitric acid/sulfuric acid solution (50 mL, v/v =1/3) at 25 °C for 8 h, then washed several times with deionized water thoroughly until the filtrate was neutral, followed by drying in an oven at 40 °C.[2] The product was then suspended in a mixture of anhydrous toluene (50 mL) and 2−cyanoethyltriethoxysilane (5 mL) under N_2 atmosphere. After refluxing for 24 h, the solid product (In−N_x−C−CN) was isolated by centrifugation, washed several times with toluene, dichloromethane and methanol, and dried under vacuum. The obtained black powder was then dispersed in ethanol (50 mL), followed by the addition of $NH_2OH·HCl$ (1.07 g) and sodium hydroxide (0.67 g).[3] After stirring at 70 °C for 24 h, the black solid was collected by centrifugation, washed several times with ethanol and deionized water, then dried under vacuum, yielding the final product In−N_x−C−R (i.e. In−N_x−C functionalized with flexible amidoxime moieties).

Materials characterization

Powder X−ray diffraction (PXRD)

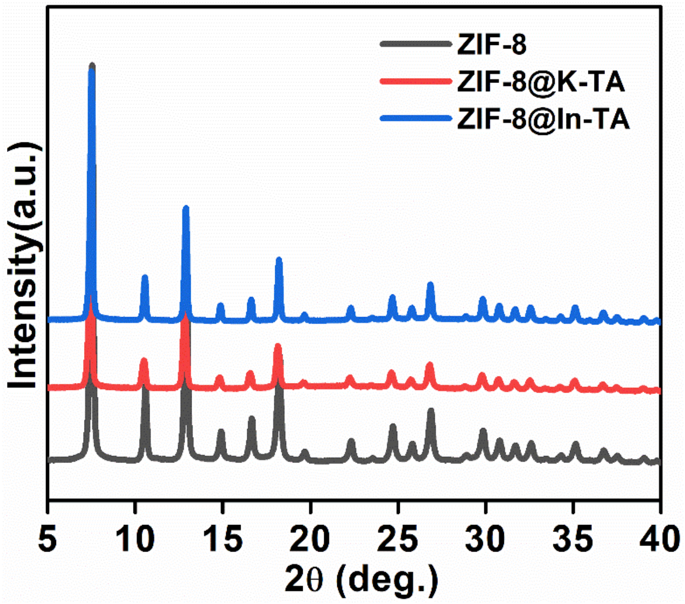

Figure S1. PXRD patterns of the as−synthesized materials.

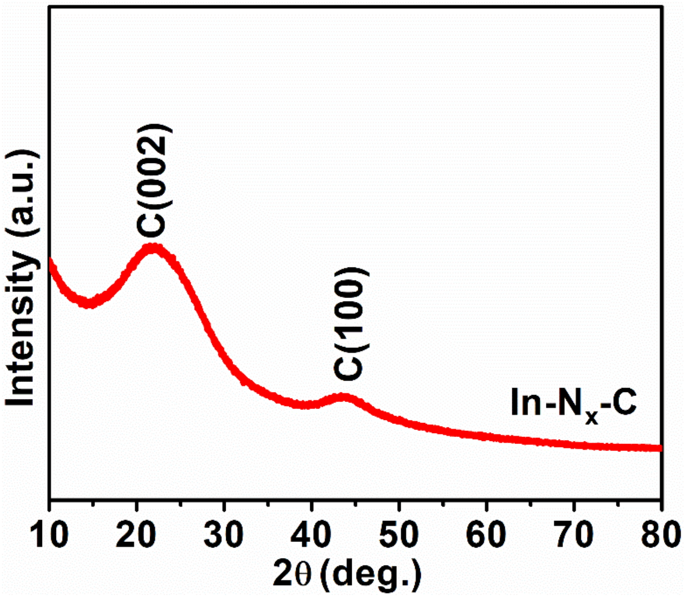

Figure S2. PXRD pattern of In−N_x−C.

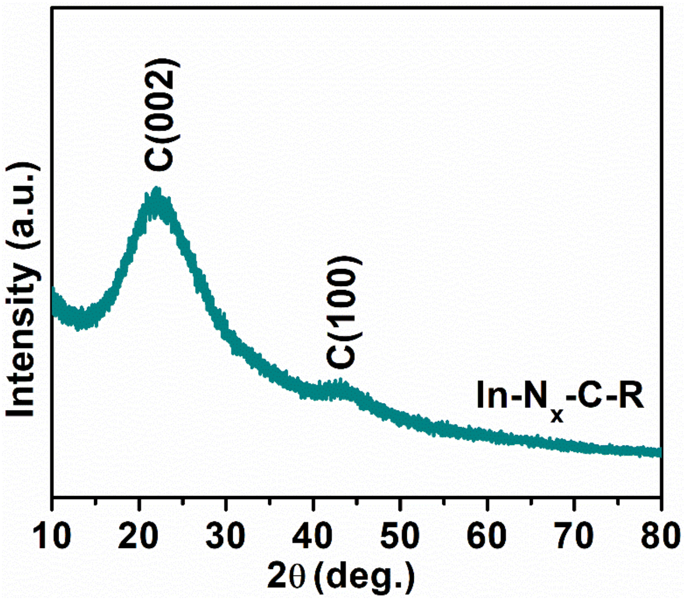

Figure S3. PXRD pattern of In−N_x−C−R.

Electron microscopy images

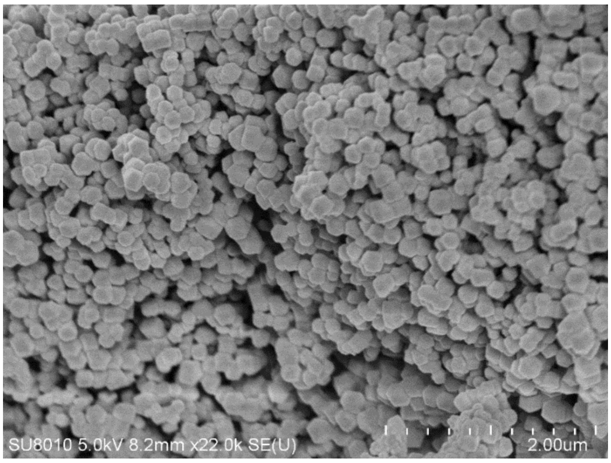

Figure S4. SEM image of ZIF−8.

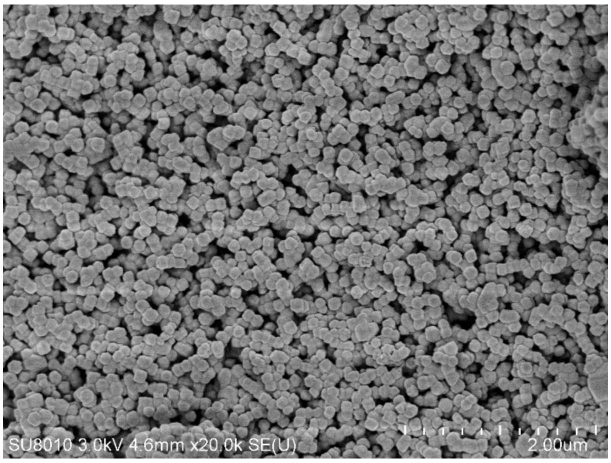

Figure S5. SEM image of ZIF−8@K−TA.

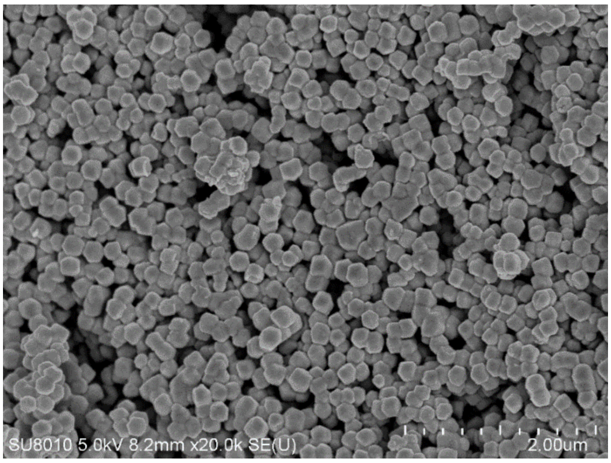

Figure S6. SEM image of ZIF−8@In−TA.

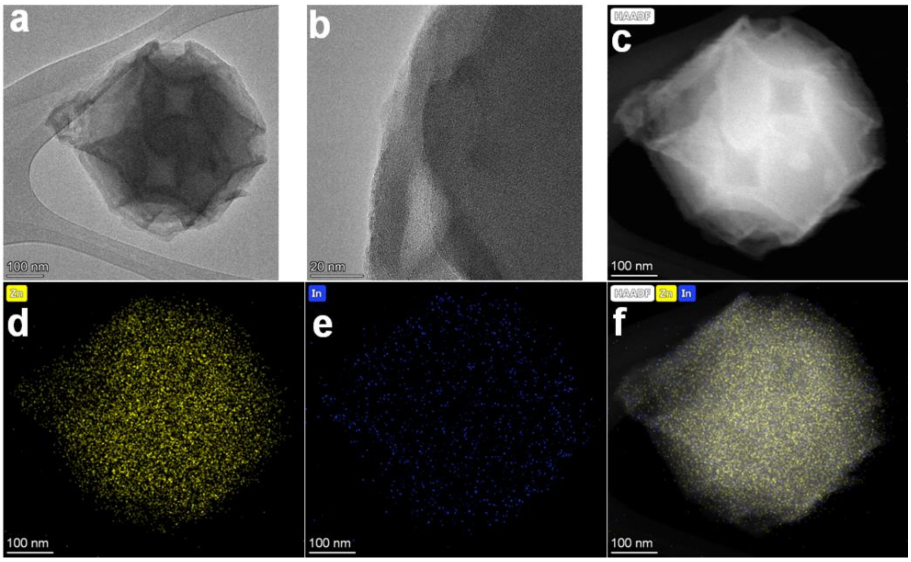

Figure S7. (a, b) TEM images of ZIF−8@In−TA. (c−f) HAADF−STEM and EDS mapping images of ZIF−8@In−TA.

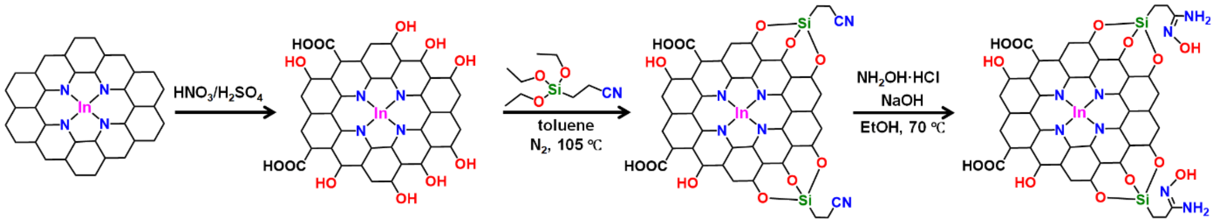

Figure S8. Schematic diagram showing the stepwise chemical transformation of In−N_x−C to In−N_x−C−R.

X−ray photoelectron spectroscopy (XPS)

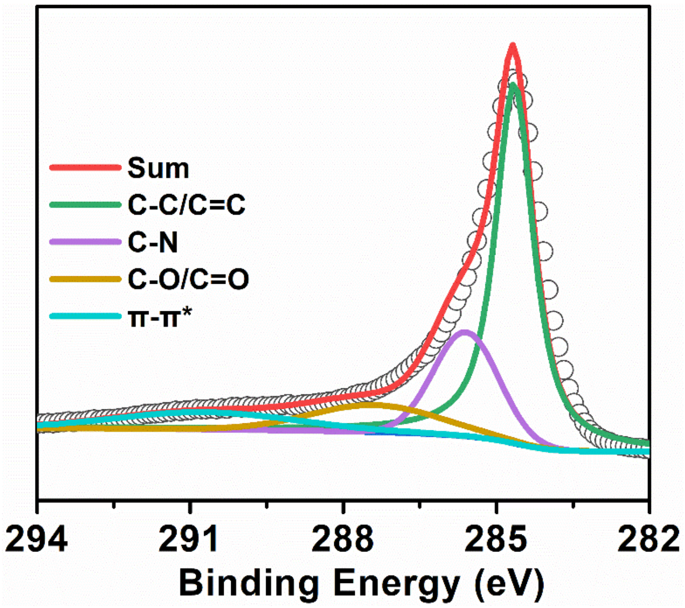

Figure S9. C 1s XPS spectrum of In−N_x−C.

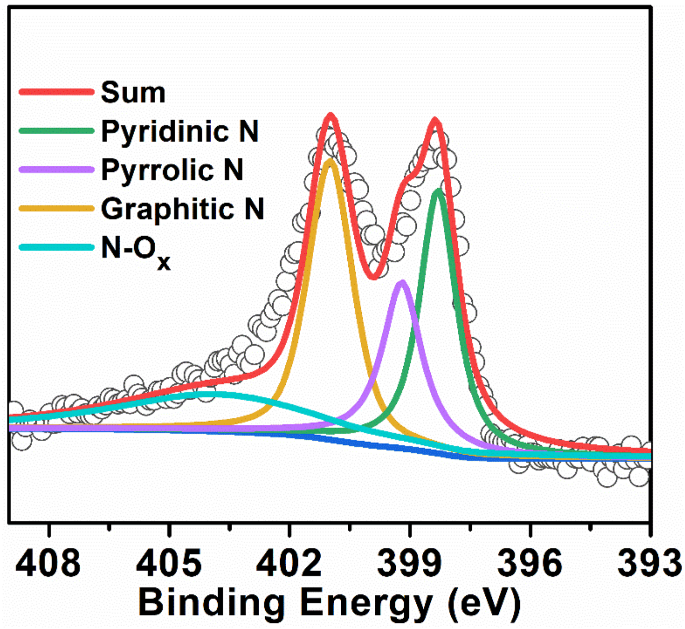

Figure S10. N 1s XPS spectrum of In−N_x−C.

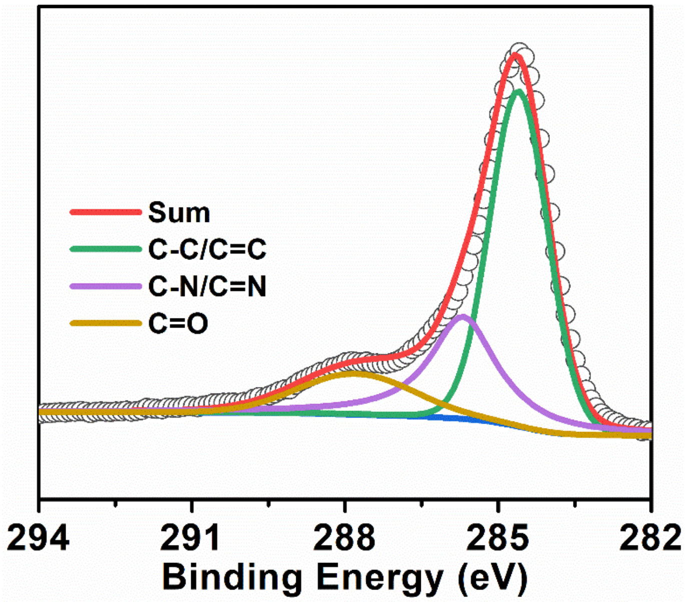

Figure S11. C 1s XPS spectrum of In−N_x−C−R.

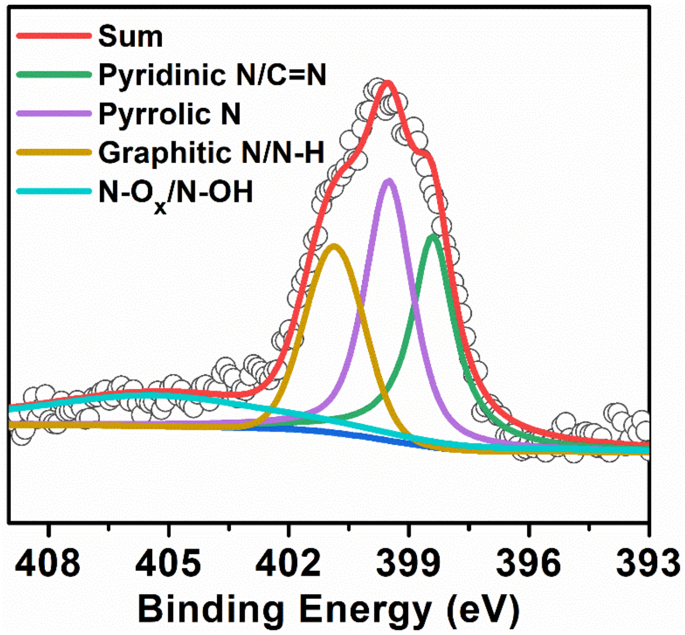

Figure S12. N 1s XPS spectrum of In−N_x−C−R.

X−ray absorption spectroscopy (XAS) measurements and analysis

In K−edge X−ray absorption spectra were collected on X−ray absorption spectroscopy beamline of SPring−8 (Japan) which was equipped with a Si(311) crystal monochromator. Fluorescence spectra were collected in transmittance mode. The energy scales were calibrated using an indium foil. The In K−edge absorption spectra were processed with the Athena and Artemis programs of the IFEFFIT package.[4]

Table S1. Summary of In K−edge EXAFS curve fitting parameters for In−N_x−C and In−N_x−C−R.

Sample	Path	*R* (Å)	*CN*	σ^2 (Å^2)	Δ*E* (eV)	*R*−factor
In−N_x−C	In−N	2.18638	4	0.00844	2.779	0.0168180
In−N_x−C−R	In−N	2.12245	4	0.00189	2.097	0.0098812

R, distance between absorber and backscattering atoms; *CN,* coordination number*;* σ^2, Debye−Waller factor to account for both thermal and structural disorders; Δ*E*, inner potential correction; *R−factor*, indicates the goodness of the fit.

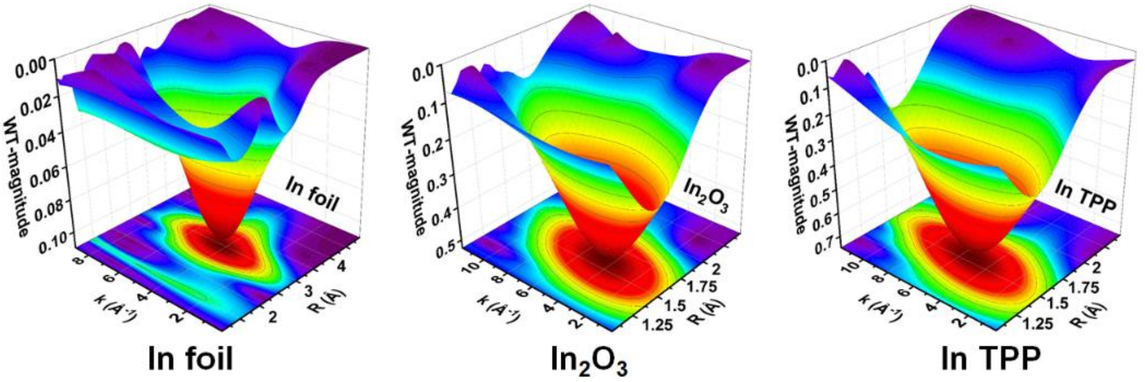

Figure S13. EXAFS−WT contour plots for In foil, In_2O_3, and In TPP.

Table S2 Summary of the indium and nitrogen contents of In−N_x−C and In−N_x−C−R

Material	In (wt.%)	N (wt.%)	Precursor	In (wt.%)
In−N_x−C	0.48	6.77	ZIF−8@In−TA	1.45
In−N_x−C−R	0.47	7.75		

Raman spectroscopy

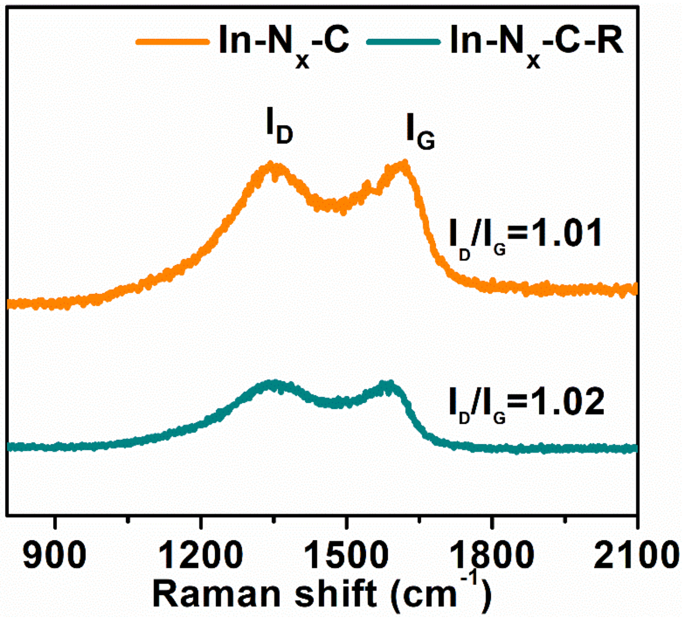

Figure S14. Raman spectra for In−N_x−C and In−N_x−C−R.

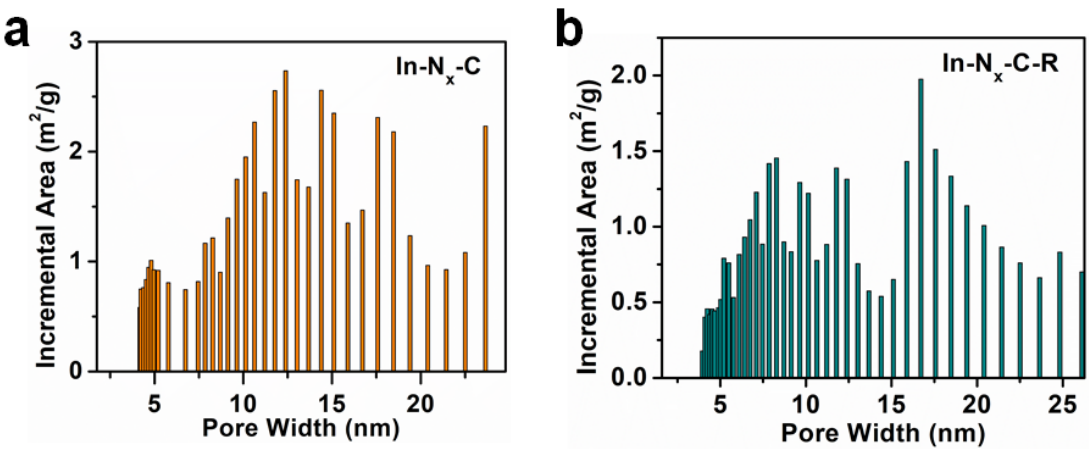

Figure S15. Pore size distributions for In−N_x−C and In−N_x−C−R.

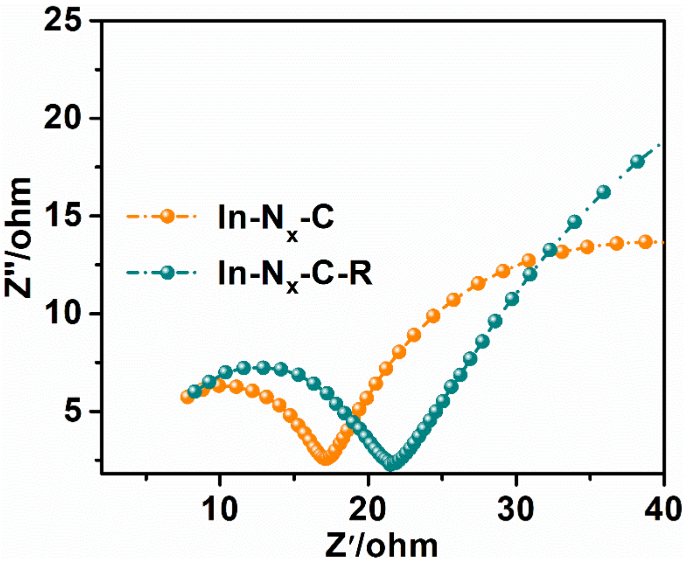

Figure S16. EIS Nyquist plots for In−N_x−C and In−N_x−C−R.

Procedures for uranium extraction

Uranium adsorption studies from uranium−spiked seawater

The adsorbents (In−N_x−C or In−N_x−C−R) were dispersed in uranyl spiked solutions of varying uranium concentration (from 0 to 100 ppm) at an adsorbent/liquid ratio of 100 mg/L. The solutions were stirred for 24 h at 25 ^oC to achieve adsorption equilibrium. Subsequently, the dispersions were filtered, and the filtrates analyzed by UV−spectrophotometry (SP−721E) using the Arsenazo III Spectrophotometric method at a wavelength of 650 nm.

The uranium uptake capacity q_e (mg/g) was calculated by following equation:

$$q_e = \frac{(C_0 - C_e) \times V}{m}$$

Where C_0 and C_e are the initial concentration and equilibrium concentration of uranium (mg/L), respectively, V is the volume of the solution (L), and m is the amount of adsorbent (g).

Langmuir and Freundlich isotherms were applied to fit the adsorption data:

$$\frac{C_e}{q_e} = \frac{1}{K_L q_m} + \frac{C_e}{q_m}$$

$$\mathrm{lg} q_e = \mathrm{lg} K_F + \frac{1}{n}\,\mathrm{lg} C_e$$

Where q_e (mg/g) and q_m (mg/g) are the equilibrium and maximum adsorption capacity of uranium, respectively, and C_0 (mg/L) and C_e (mg/L) are the initial and equilibrium concentrations of uranium, respectively. K_L is a constant in the Langmuir model, K_F and n are the Freundlich constants.

To determine the uranium adsorption kinetics, the adsorbents was dispersed in a uranyl spiked seawater solution containing 10 ppm uranium with an adsorbent/liquid ratio of 100 mg/L (the pH of the uranium−spiked seawater solutions were adjusted to ~8 using Na_2CO_3). The sample was placed in a 180 rpm shaker at room temperature, with aliquots of the dispersion being collected at regular time intervals. Subsequently, the dispersions were filtered, and the filtrates analyzed by UV−spectrophotometry (SP−721E) using the Arsenazo III Spectrophotometric method at a wavelength of 650 nm.

The pseudo−first−order kinetic model and pseudo−second−order kinetic model were applied to fit the adsorption data:

$$\ln(q_e - q_t) = \ln q_e - k_1 \mathrm{t}$$

$$\frac{t}{q_t} = \frac{1}{k_2 q_e^2} + \frac{t}{q_e}$$

Where q_t (mg/g) is the adsorption capacity of uranium at a given time, k_1 and k_2 are the rate constants for the pseudo−first−order and pseudo−second−order kinetic models, respectively.

Table S3. Summary of Langmuir and Freundlich adsorption parameters for uranium adsorption on different adsorbents in uranyl-spiked seawater.

Materials	Langmuir isotherm			Freundlich isotherm		
	K_L	q_m(mg/g)	R^2	K_F	$1/n$	R^2
In−N_x−C	0.0019	118.9	0.960	43.17	0.28	0.63
In−N_x−C−R	0.30	402.9	0.988	121.3	0.27	0.95

Table S4. Summary of sorption kinetic parameters for uranium adsorption on different adsorbents in uranyl-spiked seawater.

Materials	$q_{e,exp}$ (mg/g)	Pseudo−first−order model			Pseudo−second−order model		
		$q_{1,cal}$ (mg/g)	K_1 (min^{-1})	R^2	$q_{2,cal}$ (mg/g)	K_2 (mg/g/min)	R^2
In−N_x−C	58.48	58.44	0.16	0.999	60.01	0.004	0.996
In−N_x−C−R	95.17	91.41	0.32	0.993	94.03	0.008	0.999

Electrochemical uranium extraction studies from uranium−spiked seawater and natural seawater

All electrochemical uranium extraction tests were performed using a square wave conversion method employing alternating voltages between −5 and 0 V (using a frequency of 400 Hz during the tests) on a function/arbitrary waveform generator (UTG1005A). A graphite rod was used as the anode and In−N_x−C−R/carbon felt as the cathode. The concentrations of the uranium in the electrolyte were determined by ICP−MS. The adsorption-electrocatalytic extraction of uranium from natural seawater by In−N_x−C−R was studied under similar conditions. After uranium adsorption−electrocatalytic testing, the In−N_x−C−R/carbon felt working electrode was washed with distilled water and dried under vacuum at 40 °C. Then, the electrode was subsequently returned to the electro−reactor for further adsorption−electrocatalytic testing. Ten cycles of adsorption−electrocatalysis tests were carried out on In−N_x−C−R.

Table S5. Comparison of the performance of different adsorbents for uranium extraction from natural seawater.

Material	Conditions	Methods	Capacity for U (mg/g)	Time (d)	Capacity for U (mg/g/day)	Ref.
blank membrane without Q−CS	36 mg adsorbent	physicochemical	6.4	25	0.256	[5]
AUPM	36 mg adsorbent	physicochemical	8.78	25	0.351	[5]
Zn^{2+}–PAO	36 mg adsorbent	physicochemical	9.23	28	0.330	[3a]
DSUP fibers	10 mg adsorbent	physicochemical	17.45	3	5.82	[6]
UiO−66−3C4N	20 mg adsorbent	physicochemical	6.85	28	0.245	[7]
DNA−UEH	10 mg adsorbent	physicochemical	6.06	6	1.01	[8]
PPH−OP	10 mg adsorbent	physicochemical	7.12	21	0.339	[9]
SSUP fiber	10 mg adsorbent	physicochemical	12.33	3.5	3.523	[10]
AO−HNTs	10 mg adsorbent	physicochemical	9.01	30	0.300	[11]
MS@PIDO/Alg sponge		physicochemical	5.84	56	0.104	[12]
Tp−DBD	5 mg adsorbent	physicochemical	10.31	8	1.289	[13]
POP−oNH_2−AO	5 mg adsorbent	physicochemical	4.36	56	0.078	[14]
p(2DVB−VBC)−2 PAN	10 mg adsorbent	physicochemical	1.99	27	0.074	[15]
MIL−101−OA	100 mg adsorbent	physicochemical	4.6	5	0.92	[16]
Anti−UiO−66	5 mg adsorbent	physicochemical	4.62	30	0.154	[17]
Cp−1:12	10 mg adsorbent	physicochemical	0.55	28	0.0196	[18]
Fe−N_x−C−R	6 mg sample	adsorbent−electrocatalyst	1.2	1	1.2	[19]
MISS−PAF−1	5 mg adsorbent	physicochemical	5.76	56	0.103	[20]
PPA@MISS−PAF−1		Electrochemical method	5.4/13/16.5	14/56/90	0.385/0.232/0.183	[21]
UiO−66−AO	1 mg adsorbent	physicochemical	2.68	3	0.893	[22]
In−N_x−C−R	5 mg adsorbent−electrocatalyst	adsorbent−electrocatalyst	12.7	2	6.35	This work

U L_{III}-edge X-ray Absorption studies

Table S6. Summary of U L_{III}−edge EXAFS curve fitting parameters for In−N_x−C−R after adsorption of uranyl ions.

Samples	Path	*R* (Å)	*CN*	σ^2 (Å^2)	Δ*E* (eV)	*R*−factor
In−N_x−C−R after adsorption of uranyl	U−O_{ax}	1.78866	2.292	0.00199	8.706	0.014
	U−O_{eq1}	2.37951	3.162	0.00514	8.706	0.014
	U−O_{eq2}(N)	2.26323	2.964	0.00832	8.706	0.014

R, distance between absorber and backscattering atoms; *CN,* coordination number*;* σ^2, Debye−Waller factor to account for both thermal and structural disorders; Δ*E*, inner potential correction; *R−factor*, indicates the goodness of the fit. U−O_{ax} and U−O_{eq} represent axial coordination and plane coordination, respectively.

Table S7. Summary of U L_{III}−edge EXAFS curve fitting parameters for In−N_x−C−R after adsorption−electrocatalysis experiments.

Samples	Path	*R* (Å)	*CN*	σ^2 (Å^2)	Δ*E* (eV)	*R*−factor
In−N_x−C−R after electrocatalysis	U−O_{ax}	1.83915	2.2	0.00645	8.333	0.024
	U−O_{eq1}	2.18212	2.2	0.00299	8.333	0.024
	U−O_{eq2}	2.34436	2.2	0.00330	8.333	0.024

R, distance between absorber and backscattering atoms; *CN,* coordination number*;* σ^2, Debye−Waller factor to account for both thermal and structural disorders; Δ*E*, inner potential correction; *R−factor*, indicates the goodness of the fit. U−O_{ax} and U−O_{eq} represent axial coordination and plane coordination, respectively.

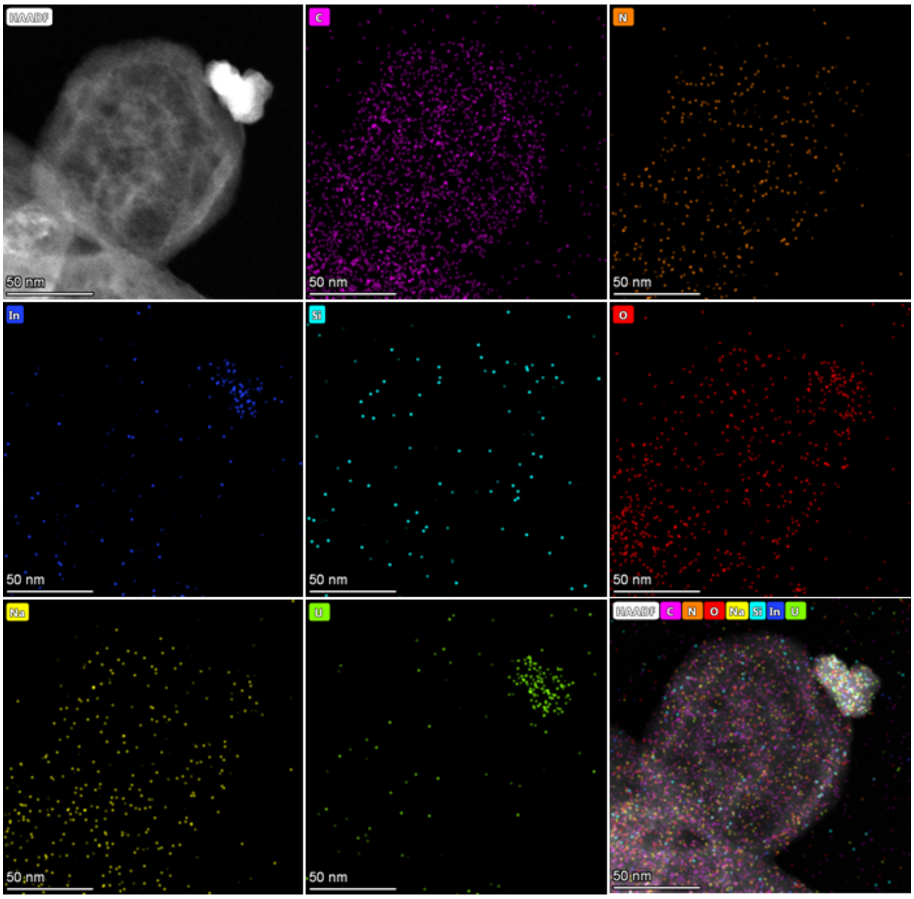

Figure S17. HAADF−STEM and corresponding EDS mapping images for In−N_x−C−R after adsorption−electrocatalysis.

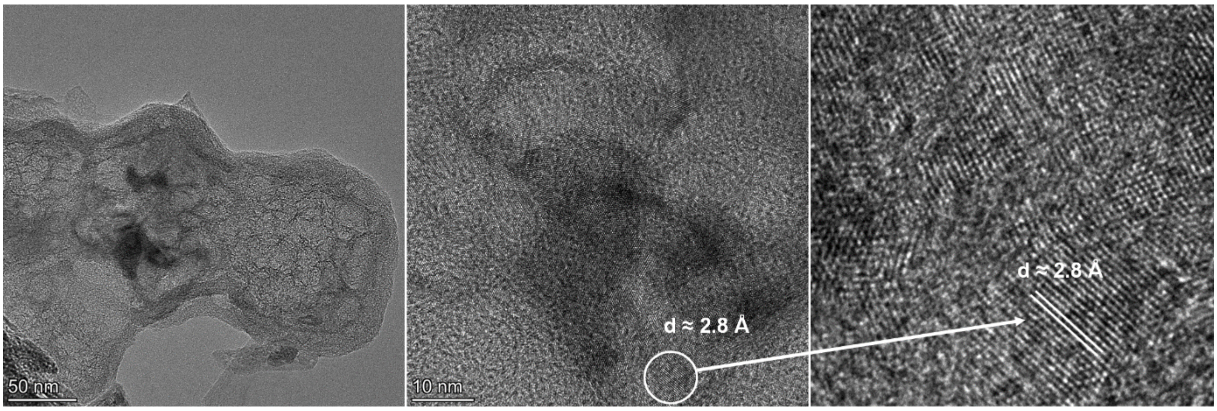

Figure S18. TEM (left) and HRTEM (right) images for In−N_x−C−R after adsorption−electrocatalysis.

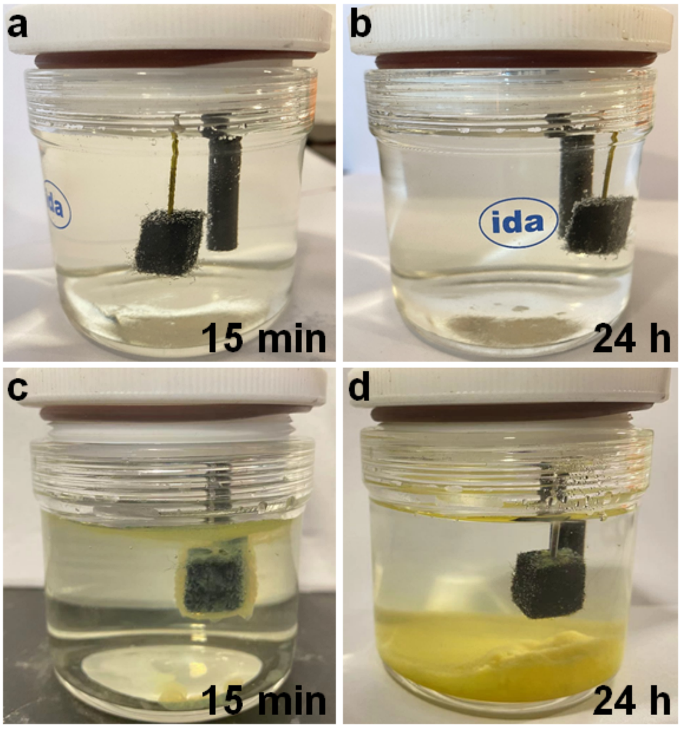

Figure S19. Photographs of ~1000 ppm uranium−spiked deionized water solution (a, b) and NaCl solution (c, d) before and after 24 h extraction using In−N_x−C−R as a catalyst.

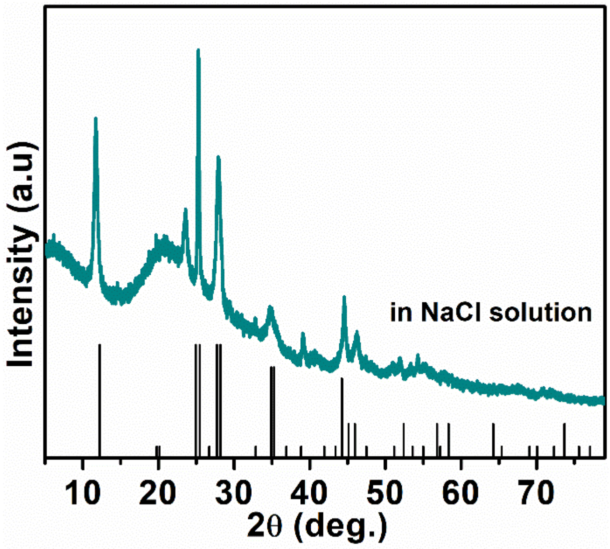

Figure S20. PXRD pattern of electrochemically−generated $Na_2O(UO_3{\cdot}H_2O)_x$ by using In−N_x−C−R as an adsorbent-electrocatalyst in NaCl solution (NaCl dissolved in deionized water).

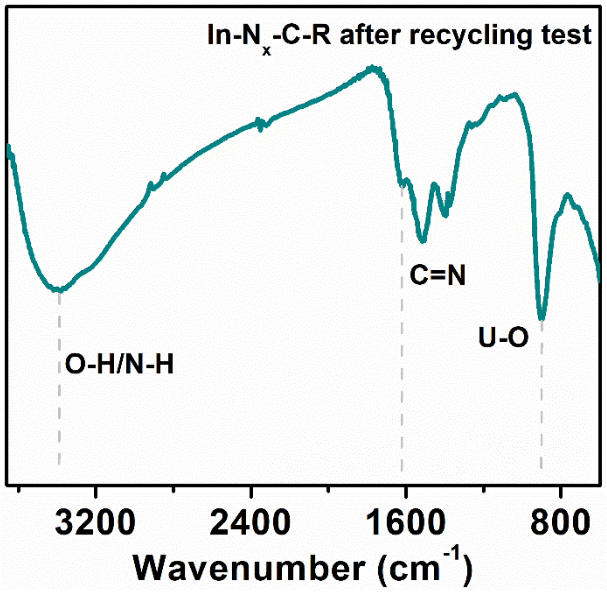

Figure S21. FT−IR spectra of In−N_x−C−R after the recycling test.

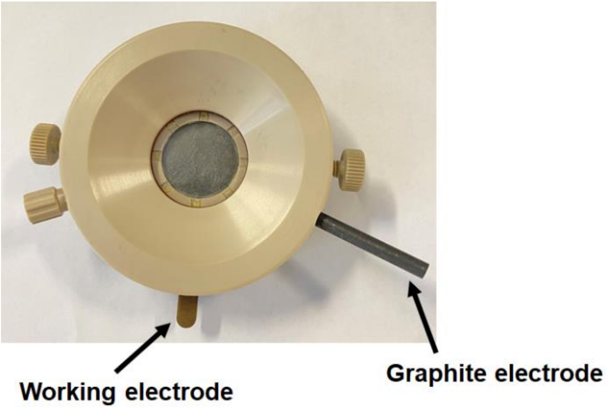

Figure S22. Photograph of *in situ* Raman cell used for investigating the formation of the $Na_2O(UO_3{\cdot}H_2O)_x$ precipitate from seawater by In−N_x−C−R. A graphite rod was used as the anode, In−N_x−C−R/carbon felt as the cathode (working electrode). The experiments were performed in uranium−spiked seawater. The *in situ* Raman cell was put onto the Raman instrument (Figure 7a in the main text). The Raman signals were recorded from 0 to 240 s (Figure 7b in the main text).

References

[1] S. R. Venna, J. B. Jasinski, M. A. Carreon, *J. Am. Chem. Soc.* **2010**, *132*, 18030-18033.

[2] a) C. Rodriguez, E. Leiva, *Molecules* **2019**, *25*, 111; b) Y.-H. Liu, Y.-Q. Wang, Z.-B. Zhang, X.-H. Cao, W.-B. Nie, Q. Li, R. Hua, *Appl. Surf. Sci.* **2013**, *273*, 68-74.

[3] a) B. Yan, C. Ma, J. Gao, Y. Yuan, N. Wang, *Adv Mater* **2020**, *32*, 1906615; b) Q. Sun, B. Aguila, L. D. Earl, C. W. Abney, L. Wojtas, P. K. Thallapally, S. Ma, *Adv. Mater.* **2018**, *30*, 1705479.

[4] B. Ravel, M. Newville, *J. Synchrotron Radiat.* **2005**, *12*, 537-541.

[5] Y. Sun, R. Liu, S. Wen, J. Wang, L. Chen, B. Yan, S. Peng, C. Ma, X. Cao, C. Ma, G. Duan, H. Wang, S. Shi, Y. Yuan, N. Wang, *ACS. Appl. Mater. Interfaces* **2021**, *13*, 21272-21285.

[6] Q. Yu, Y. Yuan, L. Feng, T. Feng, W. Sun, N. Wang, *Angew. Chem. Int. Ed.* **2020**, *59*, 15997-16001.

[7] Y. Yuan, S. Feng, L. Feng, Q. Yu, T. Liu, N. Wang, *Angew. Chem. Int. Ed.* **2020**, *59*, 4262-4268.

[8] Y. Yuan, T. Liu, J. Xiao, Q. Yu, L. Feng, B. Niu, S. Feng, J. Zhang, N. Wang, *Nat. Commun.* **2020**, *11*, 5708.

[9] Y. Yuan, Q. Yu, M. Cao, L. Feng, S. Feng, T. Liu, T. Feng, B. Yan, Z. Guo, N. Wang, *Nat. Sustain.* **2021**, *4*, 708-714.

[10] Y. Yuan, Q. Yu, J. Wen, C. Li, Z. Guo, X. Wang, N. Wang, *Angew. Chem. Int. Ed.* **2019**, *58*, 11785-11790.

[11] S. Zhao, Y. Yuan, Q. Yu, B. Niu, J. Liao, Z. Guo, N. Wang, *Angew. Chem. Int. Ed.* **2019**, *58*, 14979-14985.

[12] D. Wang, J. Song, S. Lin, J. Wen, C. Ma, Y. Yuan, M. Lei, X. Wang, N. Wang, H. Wu, *Adv. Funct. Mater.* **2019**, *29*, 1901009.

[13] W. R. Cui, C. R. Zhang, R. H. Xu, X. R. Chen, R. H. Yan, W. Jiang, R. P. Liang, J. D. Qiu, *Small* **2021**, *17*, 2006882.

[14] Q. Sun, B. Aguila, J. Perman, A. S. Ivanov, V. S. Bryantsev, L. D. Earl, C. W. Abney, L. Wojtas, S. Ma, *Nat. Commun.* **2018**, *9*, 1644.

[15] Y. Yue, R. T. Mayes, J. Kim, P. F. Fulvio, X. G. Sun, C. Tsouris, J. Chen, S. Brown, S. Dai, *Angew. Chem. Int. Ed.* **2013**, *52*, 13458-13462.

[16] H. Wu, F. Chi, S. Zhang, J. Wen, J. Xiong, S. Hu, *Micropor. Mesopor. Mater.* **2019**, *288*, 109567.

[17] Q. Yu, Y. Yuan, J. Wen, X. Zhao, S. Zhao, D. Wang, C. Li, X. Wang, N. Wang, *Adv. Sci.* **2019**, *6*, 1900002.

[18] Y. Yue, X. Sun, R. T. Mayes, J. Kim, P. F. Fulvio, Z. Qiao, S. Brown, C. Tsouris, Y. Oyola, S. Dai, *Sci. China Chem.* **2013**, *56*, 1510-1515.

[19] H. Yang, X. Liu, M. Hao, Y. Xie, X. Wang, H. Tian, G. I. N. Waterhouse, P. E. Kruger, S. G. Telfer, S. Ma, *Adv. Mater.* **2021**, 33, 2106621.

[20] Y. Yuan, Q. Meng, M. Faheem, Y. Yang, Z. Li, Z. Wang, D. Deng, F. Sun, H. He, Y. Huang, H. Sha, G. Zhu, *ACS Cent. Sci.* **2019**, *5*, 1432-1439.

[21] Z. Wang, Q. Meng, R. Ma, Z. Wang, Y. Yang, H. Sha, X. Ma, X. Ruan, X. Zou, Y. Yuan, G. Zhu, *Chem* **2020**, *6*, 1683-1691.

[22] L. Chen, Z. Bai, L. Zhu, L. Zhang, Y. Cai, Y. Li, W. Liu, Y. Wang, L. Chen, J. Diwu, J. Wang, Z. Chai, S. Wang, *ACS. Appl. Mater. Interfaces* **2017**, *9*, 32446-32451.
